# Forensic Medicine

**Published:** 1837-04

**Authors:** 


### FORENSIC MEDICINE.

### *Case of Wounds produced by Sulphuric Acid. Feigned Insanity.* Reported by M. Bopp, Advocate in Darmstadt.

THIS case is related in detached depositions, sometimes by the same, and sometimes by different witnesses. The following is a concise outline of the details.

The prosecutrix, a poor woman, stated that on the evening preceding the assault, the prisoner, a female entirely unknown to her, came to her house and solicited shelter for a short period. This the prosecutrix granted, and according to her custom, she went to bed about twelve o'clock, leaving the stranger sitting in the room. She awoke early in the morning and attended to her child, a little girl about a year and a quarter old, who slept with her. She then saw the prisoner still sitting in the room, in the same place,—a circumstance which excited her suspicion. However, she soon fell asleep again, and some time afterwards was suddenly roused up, partly by the crying of her child, and partly by a violent burning pain in her face. She started up and loudly called for assistance, in which cry the prisoner joined. A medical practitioner soon came, and it was found that some corrosive liquid, probably sulphuric acid, had been thrown over the mother and child while sleeping. The eyelids of the prosecutrix were then so swollen, that it could not be determined whether her sight had been destroyed or not. The right side of her face, her right shoulder, and left arm, had been considerably injured by the acid; while there were several severe *wounds* on the right side of the face and the right arm of the child. Proper applications were immediately made, and the further dangerous effects of the corrosive liquid thereby prevented. The healing of these injuries took place very slowly, and it was not until after fifteen weeks that the report of the patient's condition was made by the medical attendant.

There was no doubt of the liquid having been concentrated sulphuric acid, not only from an examination of the *wounds* soon after their production, but also from an analysis of some found in a bottle which was lying in the room. In defending the prisoner, an attempt was made by her counsel to show that her mind was disordered, and therefore that she ought not to be held responsible for the act. She was carefully examined, but no evidence of insanity appearing, she was condemned by the court to ten years' imprisonment for having attempted the life of the prosecutrix and her child. The execution of the sentence was deferred; since another allegation had been put in by a physician who saw her after the trial, to the effect that she was undoubtedly insane. In consequence of this, the prisoner was sent to the asylum at Hofheim, and was closely examined by the attendant physician of that establishment. He states that, at the first view of the prisoner, he suspected that she was feigning insanity. She affected to be imbecile, but her manner was obviously studied. His suspicion was confirmed when, upon enquiry, he found that, during her journey, she had talked and acted rationally enough with those who accompanied her; while he himself could obtain only trivial and unmeaning answers to his questions. The old expedient was adopted for detecting the imposture. At his next visit, the physician pretended to take compassion on her condition, remarking to the attendant, that, as the memory of the poor girl appeared to have suffered from her confinement, he should try a plan for her relief, which he had already found remarkably effectual in another case, namely, the application of a red hot iron to the back of the head; he then directed the attendant, still in the hearing of the prisoner, not to fail to have the furnace in readiness the following morning. The expedient was perfectly successful. The next morning, the memory of his patient was completely restored, and she answered all the questions put to her with the greatest readiness. During the three weeks which she afterwards remained in the asylum, the physician contrived to gain her entire confidence: she related to him her whole history, and complained of the severity of the punishment to which she had been condemned. The woman was then sent to prison, but she, a third time, pretended insanity; and on being again sent to Hofheim, she contrived one night to make her escape from the asylum. About a year afterwards, she was retaken, and then again endeavoured to make the persons around believe that she was insane The imposition, however, was too ill concealed to allow of any who saw her being deceived; and she was confined to undergo the sentence passed on her.

[*Remarks.* We are induced to call the attention of our English readers to this case for many reasons.

1. The crime, for which the prisoner was tried, is one which of late years has much increased in this country; and, to the disgrace of our criminal legislation, the law furnishes but very inadequate means for its repression, at least in England and Ireland. A case somewhat similar to the above was tried at Liverpool, in August, 1835. The prisoner, a female servant, who had been dismissed from the prosecutor's service, met her master in the street; and, without provocation, deliberately threw in his face a quantity of strong oil of vitriol, which destroyed the skin and produced serious disfigurement. She was tried on the capital charge for attempting to maim, disfigure, &c.; but the medical witnesses differed on trial as to whether a destruction of the skin by sulphuric acid could be regarded as a *wound*. The statute (9 Geo. iv. c. 31) only makes disfigurement by stabbing, cutting, or *wounding*, a felony; and it was therefore urged in the defence that this point should be clearly settled. It was reserved for the opinion of the judges; and they subsequently decided that sulphuric acid was not capable of producing a wound, within the meaning of the statute. The prisoner was therefore acquitted of the felony; but if she had used a knife, although she might not have produced so great a degree of mischief, she would have been capitally convicted. This woman was subsequently tried and punished for a misdemeanour, for which, however, not more than two years' imprisonment can be inflicted, a punishment wholly inadequate to the crime of which she had been guilty.

We are among those who consider that the definitions of medicine and surgery should be sometimes modified in their application to medical jurisprudence. It would, we think, be easy to show that no two surgical writers, however great their reputation, are agreed upon the exact signification of the word "*wound*." Some include burns under this head, while others do not; some make the essence of a wound to consist in an *external* breach of continuity, by whatever causes produced; others require that the breach of continuity should arise from a particular class of causes, and that it should take place *suddenly*. In short, there is no consistency among writers or practitioners relative to the meaning of the term. The greater number of continental medical jurists do not hesitate to classify the injuries produced by corrosive liquids among *wounds*; and it may be observed, they are as much entitled to be considered wounds as *burns*, the name which is vulgarly, but at the same time contrary to strict chemical reasoning, attached to them. But whatever difference of opinion may exist among French and German jurists on the subject, the laws of France and Germany draw no such nice distinctions as those of England. By the laws of the Codes, a prisoner is punished according to the degree of personal injury which he has inflicted, and not according to whether he has used *chemical* or *mechanical* means for its perpetration. This glaring defect in our criminal law calls loudly for removal; it either ought not to be a felony to maim or disfigure a person by maliciously stabbing, cutting, or wounding; or it should be held equally a felony for an individual to maim or disfigure another by the maliciously throwing of corrosive liquids.

2. The repeated attempts to feign insanity on the part of the prisoner constitute a singular feature in the history of this case. It is not often that we find so frequent a repetition of an imposture before individuals who have previously detected the impostor; but the prisoner in this instance feigned insanity in the face of her own former confession. That the malady was feigned, and that her object was to evade punishment, the details of the case sufficiently show; but there is one circumstance which, to our surprise, the reporter has not referred to, namely, the *probable cause* of her committing the act for which she was tried. There appears to have existed no motive whatever on her part; and we can only attribute her extraordinary conduct to a wanton and wilful feeling to injure another. There are some who hold that the mere act of a party, without any corroborating circumstances, is sufficient to indicate sanity or insanity, and to justify responsibility or the contrary. Such persons, we presume, would have pronounced this prisoner insane, and therefore irresponsible; since her act was committed without motive, and against all the common feelings of humanity. We cannot hold with this doctrine. It is true, that crime is rarely committed by a sane person without motive; but there are numerous cases in which we are unable to trace the motive; and were we, on the principle assigned, to allow of irresponsibility on these occasions, we should be assuredly overthrowing one of the great barriers established for the protection of society. On the other hand, motives for the commission of crime are sometimes discoverable in the criminal acts of persons undoubtedly insane; but this would not render it the less inhumane to make such unfortunate beings responsible for their actions, when no other evidence of insanity than that of their having been instigated by a motive, whether of revenge or avarice, could be adduced.] *Henke's Zeitschrift.* 1836.

---

*Case of Death from the Rupture of an Intercostal Artery in consequence of a Gun-shot Wound.* By Dr. Graff, Director of the Medical College of the Grand Duchy of Hesse.

The deceased, a young man, æt. fifteen, received a discharge of small shot in the thorax and abdomen, at the distance of about forty-eight paces. He instantly fell, but soon afterwards got up and ran for about six hundred paces, when he again fell exhausted. About an hour afterwards he was discovered and taken home. On examining his person, the following external injuries were observed:—1. A small round wound of the form of a middle-sized shot on the right side of the chest near the sternum, and in the interspace between the first and second ribs. From this wound, a quantity of florid blood continued to issue. 2. A wound of about the same size and shape, on the right side of the abdomen between the navel and ribs. This wound appeared to be superficial: no blood issued from it. 3. A slight contusion of a circular form on the left side of the abdomen not far from the umbilicus. The deceased died thirty-eight hours after the receipt of the wounds.

An inspection of the body was ordered to be made. On tracing the course of the wound of the chest, it was found that the substance of the pectoralis major, through which the shot had passed, was filled with thick black blood. A quantity of the same kind of blood continued to escape from the thorax through the orifice during the inspection. On laying open that cavity, the quantity found extravasated amounted to about twenty-eight ounces, the greatest extravasation being on the right side. The right lung was collapsed, occupying only about one-fourth of its cavity. There was an opening on its anterior surface at the upper part, corresponding to the external wound [1]. From this a canal was traceable for about *an inch and a quarter* into the substance of the right lung backwards: it then took a course towards the surface of the organ for about an inch and a half, and terminated in a cul de sac. At the inferior margin of the sixth rib, and at about two inches from its head posteriorly and internally, a lacerated opening of about an inch in depth was discovered. On carefully dissecting this part, the sixth intercostal artery was found torn through, and the muscular structures around were filled with blood. No foreign body was here discovered by which the wound might have been caused, nor was there any communication externally and posteriorly by which such a body might have passed out. The abdominal wound, [2] was about the size of a pea: it penetrated the abdominal cavity; but the viscera were uninjured. No shot could be discovered to account for this wound.

Dr. K—, one of those who conducted the inspection, gave the following medico-legal opinion on the cause of death. The wounds of the abdomen [2] were unimportant, and wholly unconnected with the death of the deceased. The rupture of the sixth intercostal artery, if it could be shown that this vessel had been ruptured at the time the deceased was shot, would have caused a degree of hæmorrhage highly dangerous to life. He could not, however, ascertain from the inspection how this wound in the artery had been caused, or whether hæmorrhage had really taken place from it. The wound between the first and second ribs [1] appeared to have occasioned a loss of about six pounds of blood. The death of the deceased, in his opinion, was entirely due to this wound; which was in itself *absolutely mortal*, and its consequences were not to be prevented by art.

Dr. L—, the other inspector, agreed in opinion that death was owing to excessive hæmorrhage, and to an interruption of respiration through compression from the extravasated blood. The hæmorrhage, however, according to him, arose from two sources: 1. from the wound seen externally between the first and second ribs, which may be called the *anterior* wound; and 2. from the wound internally between the sixth and seventh ribs, which may be called the *posterior* wound. He attributed both of these wounds to the small shot, which had penetrated the cavity of the chest. The hæmorrhage from the anterior wound might probably have been arrested by timely medical aid; but the posterior wound he regarded as absolutely mortal, since no means could have been here employed to stop the flow of blood. The anterior or external wound, which Dr. K— had pronounced absolutely mortal, was, according to Dr. L—, only *accidentally mortal.*

In consequence of this difference of opinion between the two inspectors, as to which wound was the immediate cause of the fatal hæmorrhage, Dr. K— was again referred to for a more precise statement of his views relative to the origin and probable effects of the posterior and internal wound. In answer to this application, Dr. K. observed, that he did not think the laceration of the intercostal artery (the posterior wound) had been caused by the shot which had penetrated between the first and second ribs (the only opening into the chest); because the wounded artery lay six or seven inches below the level of the course which the shot had taken in penetrating; and there was not the slightest communication between the canal which it had formed and the posterior wound. He attributed the laceration of the artery to the effect of disease in the lungs, such as the deposition of calcareous matter in the parenchyma, which was met with on inspection. In his opinion, the rupture of this vessel had taken place from morbid causes long before the accident; and the sole source of the blood extravasated was the anterior wound. The hæmorrhage proved fatal because there was no possibility of arresting the flow of blood in the first instance. The anterior wound therefore was by no means *absolutely*, but *accidentally mortal.*

The College was then appealed to, in order that a satisfactory report might be obtained; and the following is the substance of a very able analysis of the data upon which they had to found their decision.

"It is admitted in the report, that the sixth intercostal artery was torn through within a very short distance of its origin from the aorta, a circumstance which, notwithstanding the gradually decreasing size of the vessel and its smaller diameter at that part, would give rise to an incessant and abundant hæmorrhage. Hence death, in such a case, must speedily follow from extreme exhaustion, and from a mechanical interruption to the function of respiration. Besides, these fatal symptoms would necessarily show themselves within the first quarter of an hour after the occurrence of the rupture or laceration, from whatever cause it may have been produced. As the deceased left his home perfectly well on the morning of his death, so it follows that the laceration of the artery must have occurred between that period and the time of his falling down in an exhausted condition, most probably but a short time before he fell.

"In the opinion of Dr. K—, there must have been an aneurismal enlargement or an abscess of the intercostal artery, which burst accidentally about the very moment that the wound in the chest was received. This, however, is positively contradicted by the previously good state of health of the deceased, and still more strongly by the absence of all morbid changes indicative of these diseased conditions of the vessel at the post-mortem examination. It seems to us certain, that the laceration must have taken place but a very short time before the deceased fell, especially since, by his running six hundred paces after having received the shot, the hæmorrhage was likely to have been rendered much more profuse. It appears highly probable, if not certain, that the posterior wound must have been occasioned at the same time and by the same means as the anterior; and we have now to determine whether there be any part of the post-mortem examination opposed to the admission of this opinion. The shot which had produced the abdominal wound could not have occasioned it, since it did not traverse the diaphragm. There was no external penetrating wound at the posterior part of the chest; and indeed the only wound penetrating the cavity was that already described as situated anteriorly between the first and second ribs. Through this opening, then, the shot must have entered which produced the deep-seated laceration. From the examination of this wound during life and after death, it is clear that the canal which the shot had formed did not pass horizontally backwards, but in a direction from *above down-*

*wards.* Under these circumstances, the part at which the shot would strike posteriorly would be between the *sixth and seventh ribs.* The circumstance of no shot having been found in the neighbourhood of the wound, is no obstacle to the admission of this opinion of its origin: since it is well known that large musket bullets are often deflected from their course by a slight resistance, and lie concealed in parts remote from the wound. If this be observed with regard to such large masses of lead, *a fortiori* it would take place with small shot.

"Dr. K— has fallen into a considerable error, in stating that the wound of the intercostal artery was six or seven inches below the level of the course of the anterior wound. He seems to have forgotten, that the ribs posteriorly are much higher than they are anteriorly, and that in all well-formed skeletons a line carried *straight* backwards from the situation of the anterior wound between *the first and second ribs* would touch the upper edge of the *fifth rib* behind. Now, the distance from the upper edge of the fifth rib, posteriorly and internally, to the lower edge of the sixth, is not more than *one inch and a half.* In the report, the anterior wound is described as taking a superficial course on the right lung, after having penetrated, and terminating in a cul de sac. This description is very imperfect. The obliteration of the course of the wound through the lung backwards is easily explained by the circumstance of that organ having become so contracted in volume as to occupy only one fourth of its cavity."

The general conclusions of the College were, 1, that the deep-seated wound had been caused by the shot which penetrated, and that this wound had caused death by lacerating the sixth intercostal artery, and giving rise to profuse hæmorrhage. As no surgical assistance, even had it been at hand, could have succeeded in stopping this flow of blood, the wound was to be regarded as *absolutely mortal.* 2. That the anterior wound in the chest was, by hemorrhage from the lungs, accidentally mortal, and that it had probably had some influence in accelerating death. That the other wounds were superficial, and had no influence whatever in causing death.

[*Remarks.* This case presents several points worthy the attention of the English medical jurist.

1. The marks of violence from a gun-shot wound when small shot are used, may be very slight externally, and yet be followed by speedily fatal consequences. In the case before us the external wound was extremely small, probably not more than one shot had penetrated. Yet there can be no doubt that this shot traversed the right lung in a slanting direction, and tore asunder the sixth intercostal artery. The case consequently proves that a wound of an intercostal artery near its origin from the aorta, may prove fatal to life. The most singular circumstance connected with the wound is that the shot, after lacerating the artery and coming so closely in contact with the parietes of the chest posteriorly, should not have passed out through the skin; but experience has long since shown, that it is impossible to speculate on the course of projectiles, when they have once penetrated the cavities of the body.

2. This case also shows fully the difficulty that may sometimes exist of connecting a deep-seated internal wound with the orifice or entrance of a projectile. The canal from the collapse and shrinking of parts, especially where the projectile is small and the structure of the organ traversed by it spongy, as in the instance before us, is often highly difficult to trace. We think this sufficiently explains why the course of the shot was not discoverable. The material difference which the discovery of a communication between a superficial gun-shot wound and one which is deep-seated must make, in a medico-legal opinion respecting the cause of death, where hemorrhage may have taken place from both, is a sufficient reason for a medical jurist devoting more than ordinary attention to the performance of this part of his duty.

3. There is another point which requires to be adverted to. We are very apt, from the manner in which we acquire our knowledge of anatomy, to adopt the most erroneous views respecting the relative position of parts; and where we have not the skeleton or subject before us, we are frequently liable to fall into error respecting the course of a wound. It is not unusual to find a wound described as passing downwards, when in fact it has traversed the chest, as in the case here reported, in the antero-posterior axis. This description applies to a subject in the recumbent posture, but not to the individual as he was placed when he received the wound; an idea which we should always have before us in the medico-legal description of these injuries. The wound then in this instance passed from *before backwards*. With regard to its direction downwards or inferiorly, we have satisfied ourselves of the correctness of the opinion given by the College by the following observations. We selected four skeletons, and found, that in all a horizontal line drawn from between the first and second ribs anteriorly through the chest, touched the upper border of the fifth rib posteriorly; that in two of these skeletons, the distance from the upper border of the fifth rib posteriorly, two inches from the spine to the lower border of the sixth, was about *one inch and a quarter*, in two others *one inch and a half*. One of the skeletons was as perfect as art could make it. In this, the measurements corresponded exactly to those laid down by the College.

4. In the report we find, as usual, a great deal said about *wounds accidentally* and *absolutely mortal*. The difference in the signification attached to these terms will be sufficiently gathered from the report. The laws of some states of Germany require that this difference in wounds should always be specified by medical jurists; and accordingly, certain arbitrary rules are laid down for that purpose. To the English practitioner such a difference in the same sense as it is understood by the Germans, is wholly unimportant. He is required to state, in the event of death, merely whether the wound was the cause of death, and whether there were any circumstances which tended to aggravate its effects on the system.]

*Henke's Zeitschrift fur die Staatsarzneikunde*, 1836.

